# Efficient detection of a biomarker for infant jaundice by a europium(iii)-organic framework luminescence sensor†

**DOI:** 10.1039/c9ra08604h

**Published:** 2019-11-19

**Authors:** Ping Xu, Han-Wen Yang, Jia-Li Shi, Bo Ding, Xiao-Jun Zhao, En-Cui Yang

**Affiliations:** Key Laboratory of Inorganic-Organic Hybrid Functional Material Chemistry, Ministry of Education, Tianjin Key Laboratory of Structure and Performance for Functional Molecules, College of Chemistry, Tianjin Normal University Tianjin 300387 P. R. China encui_yang@163.com xiaojun_zhao15@163.com; Synergetic Innovation Center of Chemical Science and Engineering (Tianjin) Tianjin 300071 P. R. China

## Abstract

Efficient detection of excess bilirubin in human serum and urine is highly important for the early diagnosis of infant jaundice. A highly stable Eu(iii)-based microporous framework with bent {Eu(COO)} chains interconnected by pairs of T-shaped 4,4′-(4,4′-bipyridine-2,6-diyl)dibenzoate (bpydb^2−^) linkers, {[Eu(H_2_O)(HCOO)(bpydb)]·solvent}_*n*_ (1), was solvothermally synthesized and used as a chemical sensor for bilirubin response under clinically-applicable visible-light excitation. Due to the significant synergetic effect of the inner filter effect and photoinduced electron transfer, 1 can effectively probe trace amounts of bilirubin in aqueous solution through fluorescence decay with a strong quenching constant of 6.40 × 10^4^ M^−1^ and low detection limit of 1.75 μM. More importantly, a portable test paper made from 1 was further developed to achieve qualitative, naked-eye visualized differentiation for the biomarker in clinical applications. These interesting findings highlight the importance of the π-conjugated antenna ligand for clinically applicable Ln-MOF sensors.

## Abstract 2

A Eu(iii)–MOF sensor with intense red luminescence can effectively probe bilirubin in water through fluorescence decay, and can be fabricated into portable test papers to achieve visualized detection of bilirubin.

## Graphical abstract

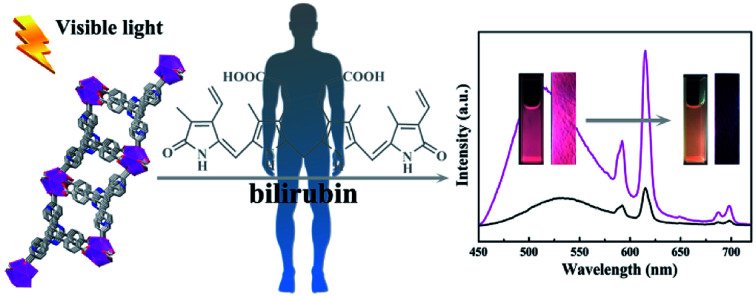

## 1 Introduction

Bilirubin, an orange-yellow metabolic breakdown product of red blood cells, plays essential physiological and pharmacological roles for anti-oxygen free radicals and anti-lipid peroxidation.^1,2^ Normal accumulated bilirubin can pass through the liver and is eventually excreted out of the body *via* bile. However, high levels of bilirubin beyond 50 μmol L^−1^ are toxic, resulting in yellowing of the skin, jaundice, brain or liver damage, and even the death of newborns.^3–8^ Thus, bilirubin serves as an important biomarker for clinically diagnosing infant jaundice or evaluating the liver function, in which a rapid and efficient response toward the excess bilirubin is of great urgency for the early diagnosis of infant jaundice.

To date, a variety of available methods including diazo,^9^ enzymatic,^10^ vanadate oxidation,^11^ electrochemical^12^ and fluorometric techniques^13^ have been consequently applied to recognize bilirubin in human serum and urine samples. Most of these approaches are time-consuming, involve complicated pre-treatment and depend on expensive instruments. Hence, simple, fast and effective assays with a highly sensitive and selective response to bilirubin are highly desirable.^14–16^ Very recently, luminescent lanthanide-based metal–organic frameworks (Ln-MOFs) have emerged as powerful chemical probes for differentiating harmful analytes due to their characteristically sharp emission, large Stokes shift, long fluorescence lifetime and quick response.^17–19^ In fact, a large number of Eu/Tb-MOFs with bright red/green emissions have been designed by far through in-corporations of versatile chromophores, post-synthetic modfications as well as host-guest self-assembly strategy.^20,21^ These versatile Ln-MOFs have exhibited remarkable capability of distinguishing toxic Pb^II^ and Hg^II^ ions,^22,23^ biomarkers for *Bacillus anthracis* spores and carcinoid tumors,^24,25^ hazardous SO_2_ and H_2_S gases,^26,27^ nitroexplosives,^28^ pharmaceutical and bio-marcomolecules^29^ through photoinduced energy transfer (PET), resonance energy transfer (RET), ground state complex formation, inner filter effect (IFE) and so on. Unfortunately, to the best of our knowledge, most of these discrimination have been triggered under the excitation of high-energy ultraviolet light, which have severely restricted their large-scale clinical on-site detection. Nevertheless, the Ln-MOF-derived sensors for bilirubin response have been scarcely reported.^13–16,30^ Herein, to gain a clinical applicable sensor for bilirubin recognization, a crystalline europium(iii)-based MOF, {[Eu(H_2_O)(HCOO)(bpydb)]·solvent}_*n*_ (1), was solvothermally generated by incorporation of a bulky π-conjugated 4,4′-(4,4′-bipyridine-2,6-diyl)dibenzoic acid (H_2_bpydb) as light-responsive ligand. As expected, possessing excellent thermal and environmental stability, 1 can emit bright red luminescent under the irradiation of the low-energy visible light (*λ*_ex_ = 432 nm), and can quickly and repeatedly distinguish trace amount of bilirubin through luminescent decay with strong quenching constant (*K*_sv_ = 6.40 × 10^4^ M^−1^) and low detection limit (1.75 μM). More importantly, the convenient test paper was further fabricated from 1, which can successfully achieve a rapidly visual detection for bilirubin by naked eyes. All these favorable advantages make 1 be a promising luminescent probe for bilirubin response. Herein, solvothermal synthesis, crystal structure, thermal and environmental stability, sensing performance and application of 1 were reported in more details.

## 2 Experimental

### 2.1 Materials and instruments

All raw reagents were commercially available and used directly without further purification. H_2_bpydb was synthesized according to the previous literature.^31^ Elemental analyses for C, H and N were carried out on a CE-440 (Leeman-Labs) analyzer. Fourier transform (FT) IR spectra were performed on an Avatar-370 (Nicolet) spectrometer with KBr pellets in the 4000–400 cm^−1^ region. Thermogravimetric analysis (TGA) was recorded on a TA Q500 thermogravimetric analyzer from room temperature to 800 °C, with a heating rate of 5 °C min^−1^ under nitrogen. Powder X-ray diffraction (PXRD) pattern was measured on a Rigaku D/max-2500 diffractometer at 60 kV and 300 mA for Cu Kα radiation (*λ* = 1.5406 Å), with a scan speed of 0.2°·min^−1^ and a step size of 0.02° in the 2*θ* ranged from 5° to 50°. Simulated PXRD pattern was calculated using single-crystal X-ray diffraction data and Mercury v1.4 program available free of charge provided by the Cambridge Crystallographic Data Center. Optical images of the samples were taken on a Leica DM6 B upright digital research microscope from Leica microsystems. Fluorescence spectra were monitored on a Fluorolog-3 fluorescence spectrophotometer from Horiba Jobin Yvon at room temperature. Luminescence quantum yield and fluorescence lifetime were obtained by an FLS920 spectrofluorometer (Edinburgh Instruments) equipped with both continuous (450 W) xenon and pulsed flash lamps. UV-vis absorption spectra were recorded with a Shimadzu UV-2700 spectrophotometer.

### 2.2 Synthesis of {[Eu(H_2_O)(HCOO)(bpydb)]·solvent}_*n*_ (1)

A mixture containing Eu(NO_3_)_3_·6H_2_O (44.6 mg, 0.1 mmol), H_2_bpydb (39.6 mg, 0.1 mmol), cyclohexanol (948.0 mg, 1.0 mL), HCl (0.4 mL, 1.0 mol L^−1^), DMF (2.0 mL), and doubly deionized water (1.0 mL) was sealed in a 23.0 mL Teflon-lined stainless-steel autoclave. The autoclave was kept at 160 °C for 72 h under autogenous pressure. After the mixture was cooled to room temperature at a rate of 5.8 °C h^−1^, brown block-shaped crystals suitable for single-crystal X-ray diffraction were obtained and separated manually (yield: 65% based on H_2_bpydb ligand). Anal. calcd for C_25.75_H_20.75_N_2.25_O_8.25_Eu (%): C, 47.90; H, 3.24; N, 4.88. Found: C, 47.60; H, 3.15; N, 5.02. FT-IR (KBr pellet, cm^−1^): 3387 (br), 3045 (w), 1676 (s), 1594 (s), 1533 (s), 1409 (s), 1384 (s), 1326 (m), 1152 (w), 1089 (w), 1014 (w), 862 (w), 816 (m), 791 (m), 746 (w), 704 (w), 628 (w), 488 (m).

### 2.3 Preparation of test paper

The filter paper was cut into strips of 0.5 cm × 1.0 cm. The dispersion of 1 (1.0 mg mL^−1^) in water was carefully dropped on these strips. The resulting strips were left at room temperature and dried in air.

### 2.4 X-ray single-crystal data collection and structure determination

Diffraction intensities of 1 were collected on a Bruker APEX-II CCD diffractometer equipped with a graphite-monochromatic Mo *K*α radiation with a radiation wavelength of 0.71073 Å by using the *φ*–*ω* scan technique at 296 K. Semiempirical multi-scan absorption corrections were applied by using SADABS.^32^ The program SAINT was used for the integration of the diffraction profiles.^33^ The structures were solved by direct methods and refined with full matrix least-squares techniques by the SHELXL-97 and SHELXS-204/7 programs.^34^ Anisotropic thermal parameters were assigned to all non-hydrogen atoms. A number of severely disordered solvent molecules existed in the MOF, which were speculated based on the electron counts calculated by PLATON software.^35^ These disorder solvents were removed by SQUEEZE during the structural refinement. The crystallographic data and selected bond lengths and angles for 1 were listed in Table 1 and Table 2, respectively.

**Table 1:** Crystal data and structure refinement for 1^a^

	1
Empirical formula	C_25_H_17_N_2_O_7_Eu
*F*_w_	609.36
Cryst size (mm)	0.18 × 0.14 × 0.13
Crystsyst	Orthorhombic
Space group	*Pbcn*
*a* (Å)	19.0174(12)
*b* (Å)	7.6764(5)
*c* (Å)	32.951(2)
*V* (Å^3^)	4810.4(5)
*Z*, *D*_c_ (g cm^−3^)	8, 1.683
*h*/*k*/*l*	−20, 22/−8, 3/−39, 35
*F*(000)	2400
*μ* (mm^−1^)	19.081
Reflections collected/unique	10 368/4258
*R*_int_	0.0752
Data/restraints/params	4258/0/317
*R*_1_ a, *wR*_2_^b^ (*I* > 2*σ* (*I*))	0.0562, 0.1370
*R*_1_, *wR*_2_ (all data)	0.0751, 0.1504
GOFonF^2^	1.007
Largest diff. peak per hole (e Å^−3^)	2.75, −1.59

a *R*_1_ = Σ(||*F*_o_| − |*F*_c_||)/Σ|*F*_o_|.

b *wR*_2_ = [Σ*w*(|*F*_o_|^2^ − |*F*_c_|^2^)^2^/Σ*w*(*F*_o_^2^)^2^]^1/2^.

**Table 2:** Selected bond lengths (Å) and angles (deg) for 1^a^

Eu(1)–O(1)	2.349(4)	Eu(1)–O(5)	2.378(5)
Eu(1)–O(2)^#2^	2.439(5)	Eu(1)–O(5)^#1^	2.453(5)
Eu(1)–O(3)^#3^	2.452(5)	Eu(1)–O(6)^#1^	2.606(6)
Eu(1)–O(4)^#3^	2.568(5)	Eu(1)–O(7)	2.416(5)
Eu(1)–O(4)^#4^	2.506(5)		
O(1)–Eu(1)–O(2)^#2^	139.47(16)	O(4)^#4^–Eu(1)–O(6)^#1^	117.91(17)
O(1)–Eu(1)–O(3)^#3^	83.29(17)	O(4)^#3^–Eu(1)–O(6)^#1^	137.22(17)
O(1)–Eu(1)–O(4)^#3^	135.02(17)	O(5)–Eu(1)–O(2)^#2^	75.21(16)
O(1)–Eu(1)–O(4)^#4^	74.39(17)	O(5)–Eu(1)–O(3)^#3^	110.53(17)
O(1)–Eu(1)–O(5)^#1^	73.39(17)	O(5)–Eu(1)–O(4)^#4^	146.87(16)
O(1)–Eu(1)–O(5)	136.77(18)	O(5)–Eu(1)–O(4)^#3^	68.16(17)
O(1)–Eu(1)–O(6)^#1^	79.55(19)	O(5)^#1^–Eu(1)–O(4)^#3^	145.92(15)
O(1)–Eu(1)–O(7)	77.33(18)	O(5)^#1^–Eu(1)–O(4)^#4^	68.07(16)
O(2)^#2^–Eu(1)–O(3)^#3^	111.12(17)	O(5)–Eu(1)–O(5)^#1^	105.53(16)
O(2)^#2^–Eu(1)–O(4)^#3^	72.31(16)	O(5)–Eu(1)–O(6)^#1^	69.06(18)
O(2)^#2^–Eu(1)–O(4)^#4^	71.80(16)	O(5)^#1^–Eu(1)–O(6)^#1^	50.62(17)
O(2)^#2^–Eu(1)–O(5)^#1^	73.72(16)	O(5)–Eu(1)–O(7)	68.54(17)
O(2)^#2^–Eu(1)–O(6)^#1^	97.13(18)	O(7)–Eu(1)–O(2)^#2^	142.33(16)
O(3)^#3^–Eu(1)–O(4)^#3^	51.99(15)	O(7)–Eu(1)–O(3)^#3^	73.79(17)
O(3)^#3^–Eu(1)–O(4)^#4^	79.15(16)	O(7)–Eu(1)–O(4)^#4^	142.69(17)
O(3)^#3^–Eu(1)–O(5)^#1^	143.66(16)	O(7)–Eu(1)–O(4)^#3^	84.78(17)
O(3)^#3^–Eu(1)–O(6)^#1^	150.87(17)	O(7)–Eu(1)–O(5)^#1^	125.38(17)
O(4)^#4^–Eu(1)–O(4)^#3^	98.43(14)	O(7)^#1^–Eu(1)–O(6)^#1^	79.56(18)

a Symmetry codes: ^#1^ 1/2 − *x*, 1/2 + *y*, *z*; ^#2^ 1/2 − *x*, *y* − 1/2, *z*; ^#3^ 1 − *x*, *y* − 1, 3/2 − *z*; ^#4^*x* − 1/2, *y* − 1/2, 3/2 − *z*; ^#5^ 1 − *x*, 1 + *y*, 3/2 − *z*; ^#6^ 1/2 + *x*, 1/2 + *y*, 3/2 − *z.*

### 2.5 Luminescent sensing of 1

Well-crashed crystalline product of 1 (30.0 mg) was immersed into the doubly distilled water (100.0 mL). The resulting mixture has been ultrasonicated for 10 min and aged for one hour to obtain the uniform dispersion of 1 (0.3 mg mL^−1^). For the luminescent sensing experiments, 200 μL freshly prepared aqueous solutions including MCl_*x*_ (M = K^+^, Na^+^, Ca^2+^, Fe^3+^, Fe^2+^ and Zn^2+^, 1.0 × 10^−3^ mol L^−1^), uric acid (UA), creatinine (Cre), creatine, glucose (Glu), ascorbic acid (AA), and bilirubin (1.0 × 10^−3^ mol L^−1^) were respectively added to the dispersion of 1 (0.3 mg mL^−1^, 3.0 mL). The emission spectra of the resultant mixture were respectively recorded in the absence and presence of different analytes mentioned above. For fluorescence titration measurements, newly prepared aqueous solution of bilirubin (1.0 × 10^−3^ mol L^−1^) was gradually introduced to the stock suspension of 1 (0.3 mg mL^−1^, 3.0 mL), and the emission spectra of the resultant mixture was respectively recorded. The interference experiments were carried out by monitoring the emission intensity of 1 at 616 nm upon stepwise introducing of bilirubin and one of the interfering substances.

### 2.6 Recycling and regeneration of 1

After each sensing experiment, the mixture was centrifugated to obtain the solid powder. The recovered powder was washed several times with distilled water and dried in air. Then, the regenerated 1 was reused in the succeeding experiments.

### 2.7 Mott–Schottky measurement

Electrochemical Mott–Schottky experiments were measured on an AMETEK Princeton Applied Research (Versa STAT 4) electrochemical workstation using complex 1/FTO combination as the working electrode, a platinum foil as the counter electrode, and a saturated Ag/AgCl/KCl as the reference electrode. The working electrode 1/FTO was prepared by dropping 50 μL of sample suspension containing 1 (1.5 mg), ethanol (0.5 mL) and Nafion (10 μL) directly onto a FTO plate. The surface area of the working electrode exposed to the electrolyte was about 1.0 cm^2^. Mott–Schottky curves of 1 and bilirubin were respectively measured in 0.2 M Na_2_SO_4_ aqueous solution (pH = 10) over the frequencies of 500, 1000, and 1500 Hz.

### 2.8 Inner filter effect (IFE) correction

The role of IFE on the suppression of the fluorescence of 1 was calculated by Parker equation. 
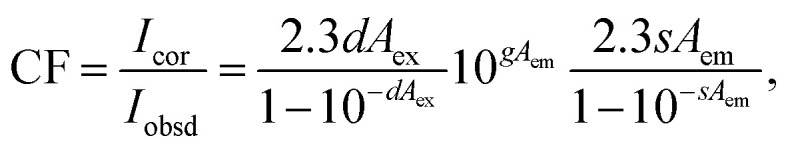
 where CF is defined as corrected factor, *I*_obsd_ refers to the measured fluorescence intensity of 1 at 616 nm under the excitation of 432 nm, *I*_cor_ is the corrected fluorescence intensity when IFE is removed from *I*_obsd_, *A*_em_ and *A*_ex_ are the absorbance of 1 having bilirubin at the excitation wavelength of 432 nm and emission wavelength of 616 nm, *s* refers to the thickness of the excitation beam (0.1 cm), *g* refers to the distance between the edge of the excitation beam and the edge of the cuvette (0.40 cm), and *d* refers to the width of the cuvette (1.00 cm).^36,37^ The observed and corrected quenching efficiency (*E*_obsd_ and *E*_cor_) were further calculated with the corresponding fluorescence intensity through *E* = 1 − *I*/*I*_0_.

## 3 Results and discussion

### 3.1 Synthesis and FT-IR spectrum of 1

Complex 1 was solvothermally synthesized at 160 °C in strongly acidic environment controlled by aqueous hydrochloric acid solution. Reaction temperature and medium were found to be important for the successful preparation of the crystalline product. Solvothermal temperature lowered than 160 °C made the crystal grain opaque, and the un-controlled medium cannot ensure the full protonation of H_2_bpydb ligand.

A broad band centered at 3387 cm^−1^ in the IR spectrum of 1 is attributed to the O–H stretching vibrations (Fig. S1†), suggesting the presence of the water molecule. A weak absorption at 3045 cm^−1^ is resulting from the stretching vibration of aromatic C–H bond from phenyl and pyridyl moieties of bpydb^2−^ ligand. As compared with the isolated H_2_bpydb molecule, an absence of a characteristic peak at 1718 cm^−1^ in 1 indicates that the carboxylic group attached on the phenyl ring is fully deprotonated. Characteristic multiple bands for asymmetric and symmetric stretching vibrations of COO^−^ are observed at 1676, 1594, 1533, 1409 and 1384 cm^−1^. Thus, the IR results are consistent with the single-crystal structural determinations.

### 3.2 Crystal structure of 1

**Fig. 1:**
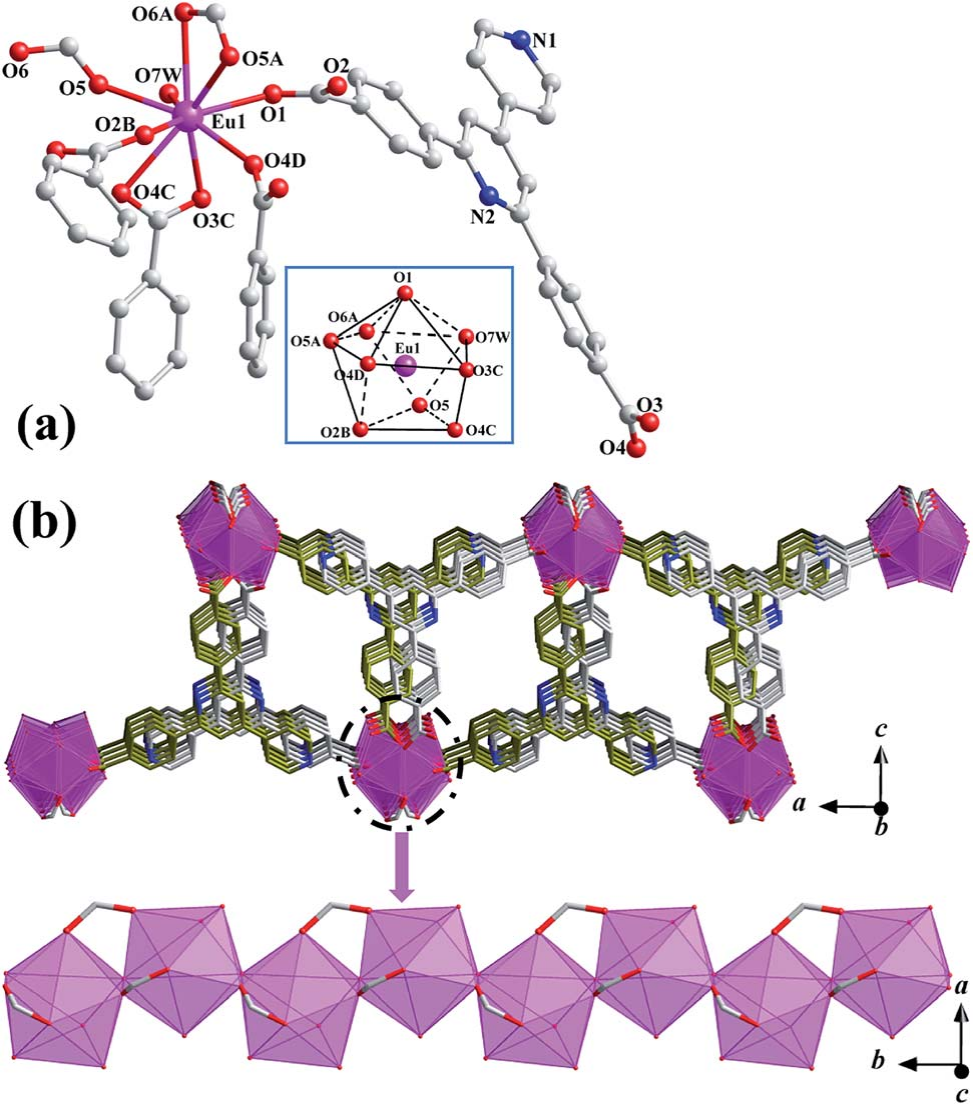
(a) Local coordination environments and polyhedron of Eu^III^ ion in 1 (H atoms were emitted for clarity, symmetry codes: *A* = 1/2 − *x*, 1/2 + *y*, *z*; *B* = 1/2 − *x*, *y* − 1/2, *z*; *C* = 1 − *x*, *y* − 1, 3/2 − *z*; *D* = *x* − 1/2, *y* − 1/2, 3/2 − *z*). (b) Microporous framework of 1 extended in the crystallographic *ac* plane.

Single-crystal X-ray diffraction analysis reveals that 1 crystallizes in the orthorhombic *Pbcn* space group (Table 1), exhibiting an isolated microporous structure with formate-propagated {Eu(COO)} chains cross-linked by T-shaped bpydb^2−^ connectors. The asymmetric unit of 1 contains one Eu^III^ ion, one doubly deprotonated bpydb^2−^ anion, one formate anion, one aqua ligand as well as disordered solvents (one water and a quarter of DMF). Notably, these solvent molecules are only identified by the PLATON squeeze program based on the electron counts. As shown in Fig. 1a, the unique Eu1 ion is in an O_9_ donor set surrounded by one terminally coordinated water molecule (O7W), eight O_carboxylate_ donors from four separate bpydb^2−^ (O1, O2B, O3C, O4C and O4D) and two individual formate anions (O5, O5A and O6A). The coordination polyhedron of the Eu^III^ ion can be described as a distorted muffin geometry with the CShM value of 1.558 evaluated by SHAPE software (Fig. 1a inset).^38^ The bond lengths of Eu–O range from 2.349(4) to 2.606(6) Å (Table 2), comparable with the previously reported Eu^III^-complexes with diverse carboxylate-derived ligands.^25,39–41^ The formate anion in 1 is solvothermally produced by the hydrolysis of DMF,^28^ adopting a bidentate chelating-bridging coordination fashion (μ_2_-η^2^:η^1^, Fig. S2†). By contrast, bulky π-conjugated bpydb^2−^ ligand is in a fully deprotonated form, exhibiting a μ_4_-η^1^:η^1:^η^2^:η^1^ manner to interact with lanthanide ions (Fig. S2†).

As shown in Fig. 1b, the separate Eu^III^ ions are held together by three-atom μ_2_-η^2^:η^1^-COO^−^ bridge, generating a zigzag chain running along the crystallographic *b* axis with the shortest intrachain Eu^III^⋯Eu^III^ distance of 4.0919(6) Å. The adjacent {Eu(COO)} chains are hold together by pairs of bpydb^2−^ connectors in a head-to-tail arrangement, resulting in a microporous framework with the nearest interchain Eu^III^⋯Eu^III^ distance of 16.0968 (10) Å (Fig. 1b). Notably, the channels of 1 are accommodate by one disordered water and a quarter of distorted DMF molecules deduced from the calculated electron number. The total potential solvent area volume and the porosity of 1 is 583.4 Å^3^ and 12.1%. These microporous frameworks of 1 are periodically stacked into a three-dimensional (3D) supramolecular network through weak interlayer C–H⋯O hydrogen-bonding interactions between the aromatic C–H of bpydb^2−^ anion and the COO^−^ anions (Table S1 and Fig. S3†).

### 3.3 Phase-purity and stability of 1

**Fig. 2:**
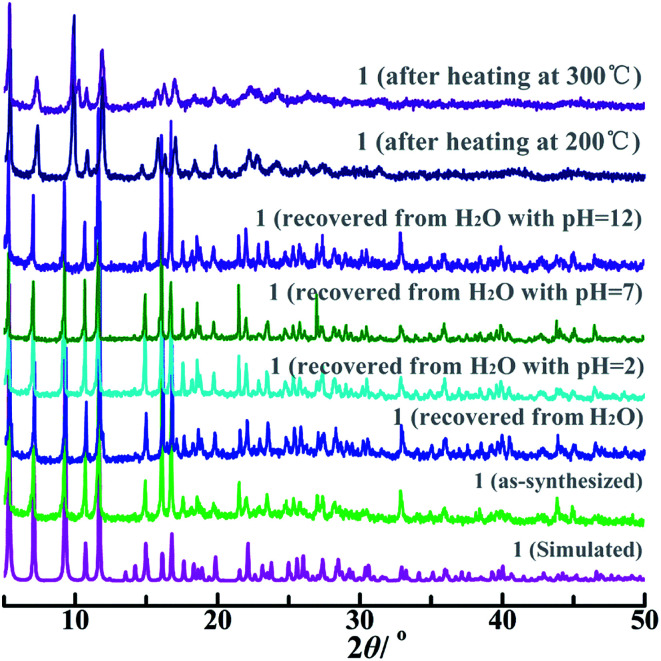
PXRD patterns of simulated and as-synthesized 1 in different external environments.

PXRD pattern of bulk as-synthesized 1 was well consistent with the simulated one produced by the single-crystal structural data (Fig. 2), suggesting high phase-purity and structural consistency of the as-prepared 1 with the single-crystal X-ray diffraction data. Compositional thermal stability and skeleton environmental stability of 1 were further examined by TGA and variable-temperature PXRD measurements (Fig. 2 and S4†). Both the disordered solvent and coordinated water molecules were released between 25 °C and 231 °C, resulting in an obviously weight-loss of 8.8% (calcd. 8.4%, Fig. S4†). The coordinated formate anion in 1 was then removed slowly upon further heating up to 422 °C, accompanying with a weight-loss of 6.7% (calcd. 7.0%). Once the temperature was higher than 500 °C, the organic ligand was partially decomposed, which was not completely finished until 800 °C.

Variable-temperature PXRD patterns of 1 after heating at 25 °C, 200 °C and 300 °C for two hours were consistent with the simulated one (Fig. 2). The environmental robustness was further checked by soaking the crystalline 1 in an aqueous solutions with pH = 2, 7, and 12 for one day or immersing the crystalline 1 in boiling water for seven day. As a result, the PXRD patterns of 1 recovered from these surroundings were good consistent with that of the as-synthesized sample. These findings reveal that 1 has the excellent composition, thermal and environmental stability, and can be potentially used as a chemical sensor.

### 3.4 Emission spectra and energy transfer of 1

**Fig. 3:**
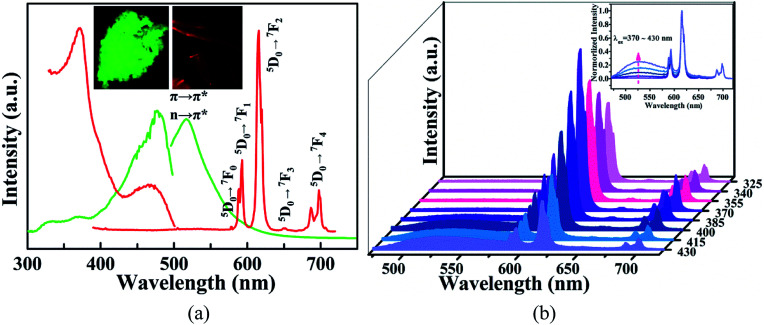
(a) Solid-state excitation and emission spectra of 1 and free H_2_bpydb molecule (inset: optical images of 1 and H_2_bpydb). (b) Excitation wavelength-dependent emission spectra of 1 in the solid-state at room temperature.

Emission spectra of solid-state 1 and free H_2_bpydb ligand were recorded at room temperature to evaluate the energy transfer pathway. Due to the intramolecular π → π* or n → π* transition, free H_2_bpydb molecule displays a broad emission band centered at 518 nm upon excitation at 477 nm (Fig. 3a). The broad emission leads to an intense green fluorescence, and can be easily naked-eye visualized under the irradiation of 365 nm UV light (Fig. 3a inset). Upon excitation at 371 nm, 1 shows five separate emission bands at 578, 593, 616, 650, and 698 nm corresponding to the characteristic ^5^D_0_ → ^7^F_*J*_ (*J* = 0–4) transitions of Eu^III^ ion. The intense emission make 1 emit a naked eye-visualized red signal (Fig. 3a inset) with the quantum yield (QY) and lifetime (*τ*) of 5.38% and 477 μs monitored at 371 nm and 616 nm. Notably, the emission intensity ratio of the electric-dipole ^5^D_0_ → ^7^F_2_ (616 nm) and magnetic-dipole ^5^D_0_ → ^7^F_1_ (593 nm) transitions is about 2.8, indicating that the Eu^III^ ion in 1 occupies a low-symmetry coordination site with no inversion center^42^ and is well consistent with the results of single-crystal X-ray structural analysis. The ^5^D_0_ → ^7^F_2_ transition has a large intensity comparable to that of the ^5^D_0_ → ^7^F_4_ transition, suggesting that the intensity parameter *Ω*_λ_ is in a weak polarizable environment.^43–45^ Additionally, the disappearance of the ligand-centered emission in 1 demonstrates that the bpydb^2−^ anion acts as a good antenna ligand to efficiently sensitize the luminescence of the Eu^III^ ion.

To deep insight into the energy transfer pathways between the bpydb^2−^ ligand and Eu^III^ ion, excitation wavelength-dependent emissions of 1 were measured at room temperature, together with the *τ* values at 616 nm and excitation wavelength-dependent QY measurements. As shown in Fig. 3b, the emission intensity of 1 at 616 nm (*I*_616_) gradually increases with the increasing excitation wavelengths from 325 to 370 nm, while the QY decreases monotonously and *τ* values varies only slightly (Table S2†). Once the excitation light is beyond 370 nm, the *I*_616_ declines dramatically, accompanying with an appearance of a new broad band centered at 518 nm and apparently shortened lifetime at 616 nm (Fig. 3b inset and Table S2†). These observations reveal that the different energy transfer pathways occur between Eu^III^ ion and bpydb^2−^ ligand. With the gradual decrease of the excitation energy, the *S*_1_ → *S*_o_ transition of the bpydb^2−^ happens, accompanying the incomplete energy transfer from *T*_1_ of the ligand to the ^5^D_0_ state of the Eu^III^ ion for the decreased fluorescence intensity at 616 nm.

Since the UV light is strongly scattering and can significantly mutate cells and tissues responsible probably for premature aging or skin cancer, the low-energy excitation in the visible region is highly desirable and particularly significant for the clinical applications of the probes. Due to the favorable emission intensity of 1 in the visible region and the maximum absorption wavelength of bilirubin, 432 nm was then selected as the optimal excitation energy for the sensing investigations of 1. It should be mentioned that the QY and *τ* values of 1 is 2.85% and 413 μs upon excitation at 432 nm (Table S2 and Fig. S5†), slightly smaller than those of 1 excited at the optimal excitation wavelength (*λ*_ex_ = 371 nm).

### 3.5 Selective sensing of 1 towards bilirubin

**Fig. 4:**
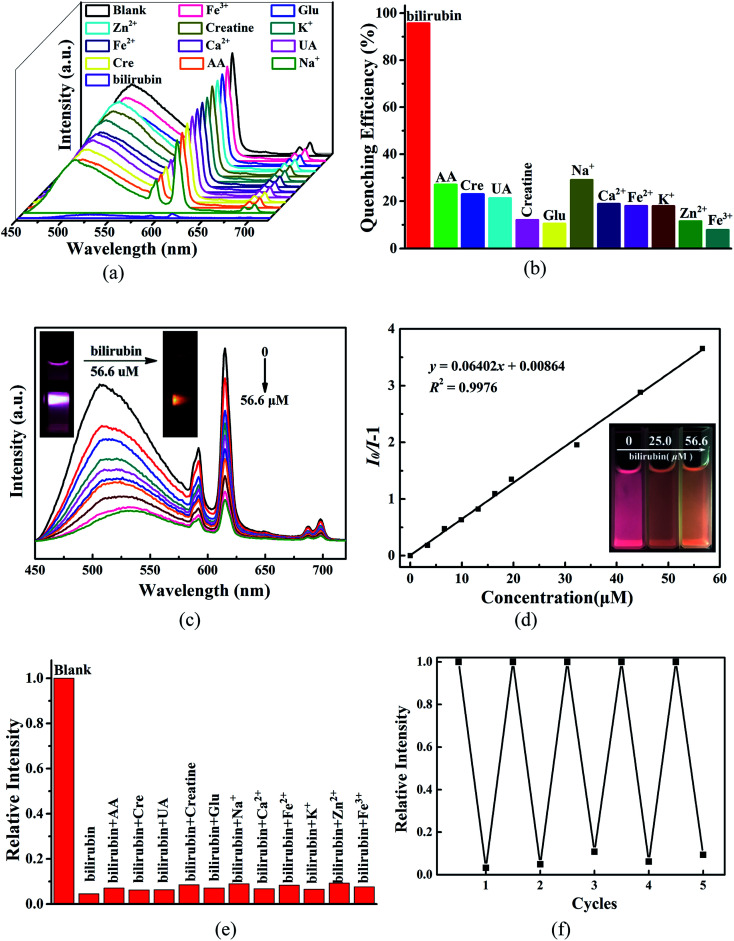
(a) Emission spectra of 1 in the absence and presence of different components of urine. (b) Luminescent intensity of 1 suspension at 616 nm in the presence of different substances of urine. (c) Emission spectra of 1 in the presence of different concentrations of bilirubin. (d) Plot of (*I*_0_/*I* − 1) *vs.* [bilirubin]. (e) Fluorescence response of 1 upon addition of bilirubin together with one of other interfering chemicals. (f) Recycling of 1 implemented with bilirubin aqueous solution.

The intense red emission, robust stability and low-energy excitation motivate us to investigate the responsive ability of 1 to bilirubin in water system. Upon excitation at 432 nm, the *I*_616_ of 1 decrease to some different degree in the presence of different substances in the urine (Fig. 4a). Quenching efficiency of 1 defined as (*I*_0_ − *I*)/*I*_0_ × 100% is 95.7% by bilirubin, remarkably higher than the other components existing in the urine (7.9–27.0%, Fig. 4b). Thus, 1 can selectively distinguish bilirubin in the uniform dispersion through luminescent quenching. To quantitatively evaluate the quenching constant (*K*_sv_) by bilirubin, luminescence titration was carried out by incremental addition of bilirubin stock solution into the dispersion of 1. As illustrated in Fig. 4c, the *I*_616_ declines gradually with the gradually increasing bilirubin. When the concentration of bilirubin is 3.32, 19.6 and 56.6 μM, the *I*_616_ of 1 is 15.4%, 57.4% and 78.5% of its initial intensity. Moreover, apparent color change of the dispersion system can be easily observed by the naked eyes under the irradiations of 432 nm (Fig. 4c inset) and 365 nm of portable UV lamp (Fig. 4d inset). The plot of *I*_0_/*I vs.* [bilirubin] is straightly linear within the whole concentration range examined herein, suggesting a static quenching mechanism. The plot of *I*_0_/*I vs.* [bilirubin] can be well fitted to the Stern–Volmer (S–V) equation (*I*_0_/*I*) = *K*_sv_[M] + 1, in which *I*_0_ and *I* are luminescent intensity of 1 in the absence and presence of bilirubin, respectively; [M] is the molar concentration of bilirubin, and *K*_sv_ is the quenching constant (M^−1^).^41^ The best fit affords *K*_sv_ = 6.40 × 10^4^ M^−1^ and an excellent correlation coefficient *R*^2^ = 0.9976. The *K*_sv_ of 1 towards bilirubin is slightly lower than that of the sole MOF sensor UIO-66-PSM (Table 3).^30^ Moreover, as compared with UIO-66-PSM, 1 has two additional advantages: simple preparation (solvothermal synthesis *vs.* post-synthetic modfication) and visible light excitation (*λ*_ex_ = 432 nm *vs. λ*_ex_ = 340 nm). Additionally, although possessing larger *K*_sv_ values than that of 1 (Table 3), the sensing of both BAMD and HSA-AuNCs towards bilirubin occurred under the excitation of UV light, which has severely restricted their clinical application and can not be easily observed by naked-eye.

**Table 3:** Comparison for the reported luminescence probes for bilirubin

Sensors	Linear range/μM	LOD/nM	*K*_sv_/M^−1^
BAMD^3^	1 × 10^−6^–500	0.0028	3.3 × 10^10^
HSA-AuNCs^7^	1–50	248 ± 12	5.5 × 10^5^
Nanosheets-tta/PS^13^	0–60	41	—
S,N-CDs/Fe(iii)^15^	0.2 × 10^−3^–2 × 10^−3^	0.12	—
BSA-CuNCs/Fe^3+^ (ref. 16)	0.1 × 10^−6^–0.1	6.62	—
UIO-66-PSM^30^	0–500	0.00059	8.95 × 10^4^
{[Eu(H_2_O)(HCOO)(bpydb)]·solvent}_*n*_	0–56.6	1750	6.40 × 10^4^

Limit of detection (LOD) is also an important parameter that describes the sensing ability of the sensor. The LOD of 1 towards bilirubin is 1.75 μM calculated from the equation LOD = 3*σ*/*k*, wherein *σ* is the standard deviation of five separate blank measurements and *k* is the slope extracted from the plot of *I vs.* [bilirubin] (Fig. S6†). The LOD of 1 is much lower than the safety content of bilirubin in the human serum, but much higher than that of UIO-66-PSM and other types of sensors (Table 3).^30^ Therefore, much efforts should be specially paid on the improvement of the LOD, in which the enhancement of *σ* is much more important than that of *k*.

To evaluate the sensing specificity of 1 towards bilirubin in the practical applications, the potential influences from the interfering biomolecules and metal cations in the urine on the sensing behavior were considered. As demonstrated in Fig. 4e, the fluorescence quenching effect of 1 by bilirubin is almost comparable to those caused by bilirubin and one of the interfering substances including UA, Cre, AA, Creatine, Glu, Na^+^, Fe^2+^, Zn^2+^, K^+^, Ca^2+^ and Fe^3+^. These observations show that 1 can discriminate bilirubin in a highly selective and sensitive manner without being disturbed by other components in the urine sample.

The recyclable performance of 1 on the response of bilirubin was also checked. After at least five continuous sensing recycles, both the luminescence intensity and PXRD pattern of the recovered 1 were almost the same as those of the original sample (Fig. 4f and S7†). Response rate and discrimination stability of 1 to bilirubin in physiological conditions were also explored. For the physiological pH-responsive behavior, 1.0 mg of 1 was added to 3.0 mL water with pH = 4–8 adjusted by HCl or NaOH and the emission spectra of 1 is the similar to each other and the *I*_616_ is thus pH-independent (Fig. S8†). Moreover, the fluorescence intensity of 1 decreases rapidly within two seconds upon contacting with bilirubin in aqueous solution with pH = 7.4, and the decayed intensity can keep constant for two min (Fig. S9†). Therefore, 1 can serve as a superior chemical sensor for bilirubin with multiple advantages of fast response, good recycling, robust stability, high selectivity and sensibility.

### 3.6 Sensing mechanism

**Fig. 5:**
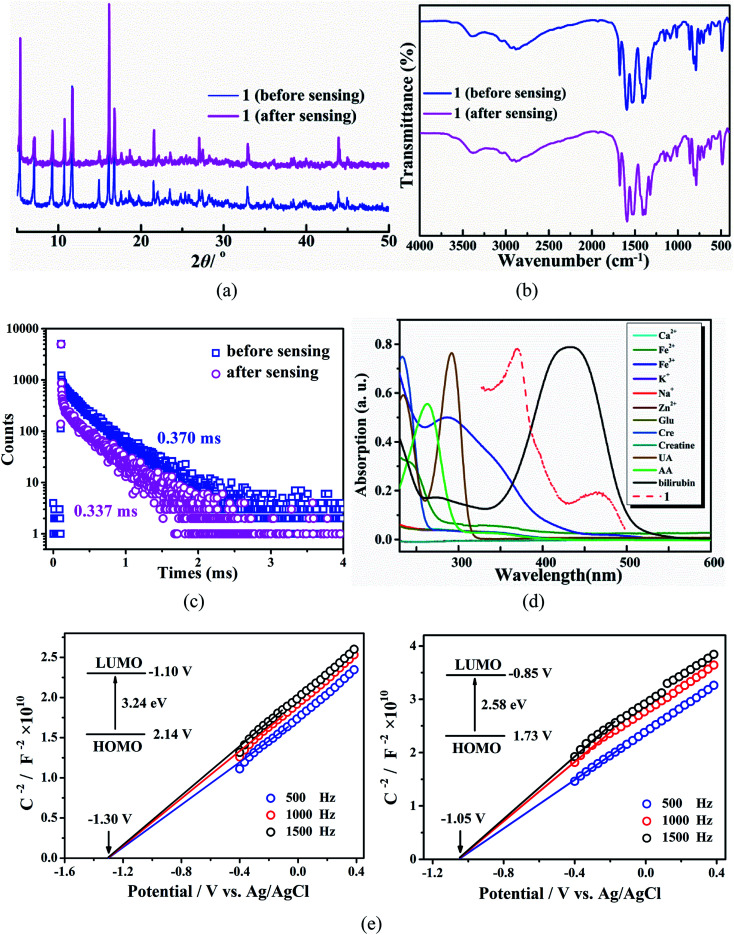
(a) PXRD patterns of 1 before and after bilirubin sensing. (b) FT-IR spectra of 1 before and after bilirubin response. (c) Time-resolved decay curves of 1 at 616 nm in the absence and presence of bilirubin. (d) UV-vis absorption spectra of different bio-molecules and metal ions in aqueous solution and excitation spectrum of 1. (e) Mott–Schottky curves and energy band positions of 1 and bilirubin.

To deep understand the responsive mechanism, PXRD patterns, FT-IR spectra and fluorescence lifetimes of 1 were investigated before and after bilirubin sensing, together with the UV-vis absorption of all the examined analytes. Both PXRD patterns and FT-IR spectra of 1 remain consistent before and after the sensing experiments (Fig. 5a and b), confirming the quenching is not caused by the framework collapse, ion exchange, as well as the strong interactions between 1 and bilirubin. Additionally, the *τ* value of 1 at 616 nm is only slightly shortened from 370 to 337 μs after addition of bilirubin (180 μL, 1.0 × 10^−3^ mol L^−1^, Fig. 5c), suggesting that a static quenching mechanism occurred during the discrimination process, not any Förster resonance energy transfer (FRET).^46^

To further confirm the possibility of the inner filter effect and photoinduced charge transfer (PET) between 1 and bilirubin, the UV-vis absorption spectra and Mott–Schottky curve were measured, respectively. As shown in Fig. 5d, bilirubin displays a broad absorption centered at 432 nm among the UV-vis absorption spectra of the twelve analytes examined herein, which overlaps well with the excitation spectrum of 1. Therefore, both bilirubin and 1 can effectively absorb the excitation energy, and the more favorable absorption of bilirubin towards the excitation light than 1 can weaken the energy transfer efficiency from bpydb^2−^ to Eu^III^, resulting in the quenching of characteristic luminescence of Eu^III^ ion in 1. To eliminate the influence of inner filter effect (IFE) on the fluorescent decrease, the IFE correction was further considered with through Parker equation. The observed and corrected quenching efficiency (*E*_obsd_ and *E*_cor_) were respectively obtained as 78.5% and 34.4% (Table S3, Fig. S10 and S11†). Thus, the quenching cause by IFE and PET mechanism are about 44.1% and 34.4%, respectively.

The PET process can be further analyzed by comparing the energy levels of 1 and bilirubin. The band gap extracted from the UV-vis diffuse reflectance spectra is 3.24 ev and 2.58 eV for 1 and bilirubin (Fig. S12†). The flat band potential determined from the intersection is approximately −1.30 V (*vs.* Ag/AgCl) and −1.10 V (*vs.* NHE) for 1 as well as −1.05 V (*vs.* Ag/AgCl) and −0.85 V (*vs.* NHE) for bilirubin (Fig. 5e). According to their band gap values, the valence band potential is calculated to be 2.14 V and 1.73 V (*vs.* NHE) for 1 and for bilirubin. The work function (*E*) of 1 and bilirubin *vs.* vacuum can be obtained using equation *E* (eV) = −4.5 − *E*_NHE_ (V).^47^ Considering the conduction band potentials of 1 and bilirubin are −3.40 eV and −3.65 eV, a direct PET from 1 to bilirubin can easily occur, responsible for the decay of the 1 at 616 nm. Thus, the competition of absorption between bilirubin and ligand in 1 and PET from 1 to bilirubin should be jointly responsible for the selective and sensitive detecting of bilirubin in water system.

### 3.7 Fluorescence test paper of 1

**Fig. 6:**
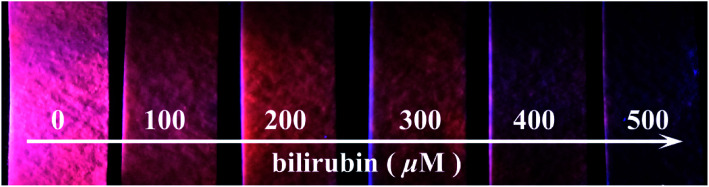
Optical images of 1-coated test papers in the absence and presence of aqueous bilirubin solution under the irradiation of 365 nm UV lamp.

A portable and economic test paper was designed to advance the bilirubin response of 1 more simple and more feasible. The as-prepared test papers were soaked in aqueous bilirubin solutions with different concentrations for 30 second and then exposed under the UV lamp of 365 nm. As shown in Fig. 6, the luminescent color of the paper exhibited intense red in the absence of bilirubin observed by the naked eyes. With the increasing amounts of the bilirubin from 0 to 500 μM, the color of the test paper changed quickly and become darker and darker upon exposure of the UV lamp, which are suitable for hands on use. Thus, the portable test paper can be used as a promising luminescent sensor for the diagnosis of jaundice detection in a simple and quick manner.

## 4 Conclusions

A highly robust europium(iii)–organic framework with bent chains cross-linked by π-extended 4,4′-(4,4′-bipyridine-2,6-diyl)dibenzoate connectors was solvothermally generated and structurally characterized. Resulting significantly from the inferior absorption of antenna ligand towards the irradiated light and photoinduced electron transfer from the sensor to bilirubin, the framework with intense red luminescent can quickly and repeatedly detect bilirubin in water system through fluorescence decay with strong quenching constant up to 6.40 × 10^4^ M^−1^ and low LOD down to 1.75 μM. More interestingly, a portable test paper prepared from the sensor was developed, providing visual detection of bilirubin by naked eyes in the feasible applications. These interesting results demonstrate the significance of the antenna ligand with π-conjugated skeleton on the promising luminescent sensors in the clinical diagnosis of jaundice.

## Conflicts of interest

There are no conflicts to declare.

## Supplementary Material

RA-009-C9RA08604H-s001

RA-009-C9RA08604H-s002
