# Supplementary material for: Efficient detection of a biomarker for infant jaundice by a europium(iii)-organic framework luminescence sensor
Source: RSC Adv. 2019 Nov 19;9(64):37584–93. doi: 10.1039/c9ra08604h (PMC9075806; doi:10.1039/c9ra08604h)
Supplement: RA-009-C9RA08604H-s001 [file RA-009-C9RA08604H-s001.pdf]

Electronic Supplementary Material (ESI) for RSC Advances.
This journal is © The Royal Society of Chemistry 2019

Supporting information

Efficient detection of biomarker for infant jaundice by a luminescent europium(III)-organic framework sensor

Ping Xu[a], Han-Wen Yang[a], Jia-Li Shi[a], Bo Ding[a], Xiao-Jun Zhao[a, b,*], En-Cui Yang[a,*]

[a] *Key Laboratory of Inorganic-Organic Hybrid Functional Material Chemistry, Ministry of Education, Tianjin Key Laboratory of Structure and Performance for Functional Molecules, College of Chemistry, Tianjin Normal University, Tianjin 300387, P. R. China. E-mail: encui_yang@163.com, xiaojun_zhao15@163.com*

[b] *Synergetic Innovation Center of Chemical Science and Engineering (Tianjin), Tianjin 300071, P. R. China*

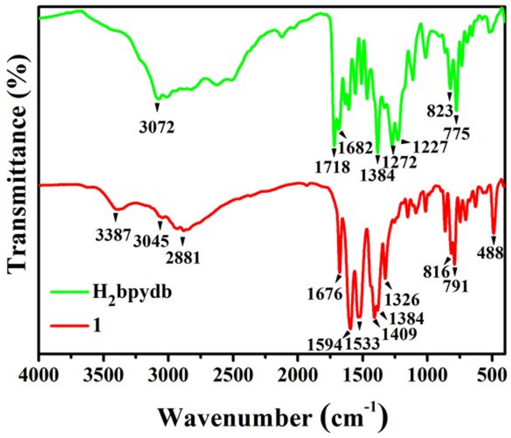

Fig. S1 FT-IR spectra of **1** and H_2bpydb ligand.

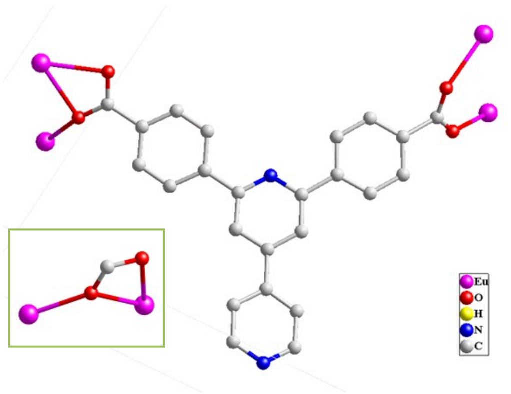

Fig. S2 Binding modes of bpydb^{2-} and formate ligands (insert) in **1**.

Table S1 Selected hydrogen-bonding parameters for **1**[a]

D–H···A	*d* (D–H)	*d* (H···A)	*d* (D···A)	*θ* (D–H···A)
C16–H16···O6[#1]	0.93	2.37	3.251(10)	158
C20–H20···O3[#2]	0.93	2.54	3.195(9)	128

[a] Symmetry codes: [#1] 1/2 + *x*, 1/2 – *y*, 1 – *z*; [#2] 3/2 – *x*, –1/2 + *y*, *z*.

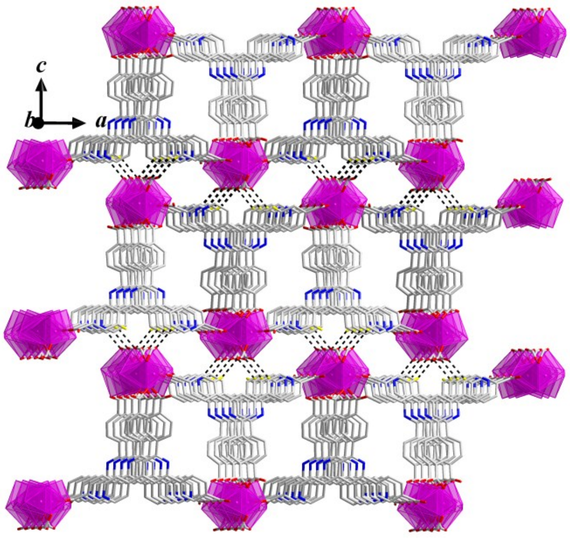

Fig. S3 Three-dimensional supramolecular structure of **1**.

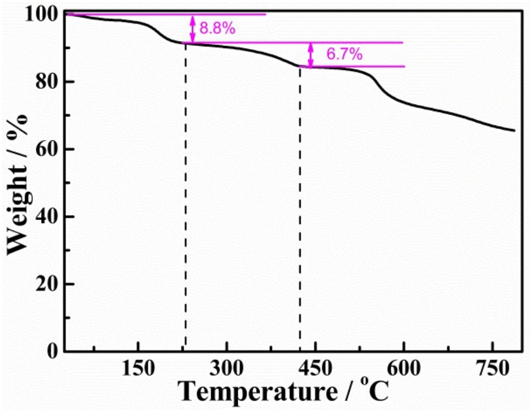

Fig. S4 TGA curve of **1**.

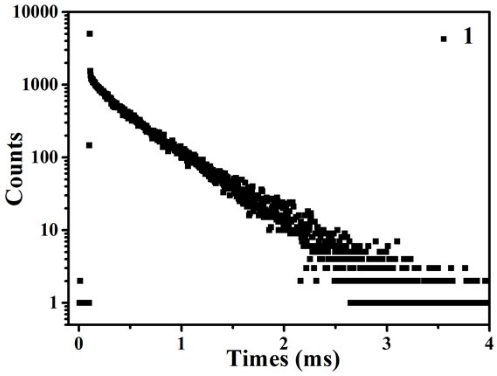

Fig. S5 Fluorescent lifetime of **1** monitored at 616 nm in the solid state upon excitation at 432 nm.

Table S2 Excitation wavelength-dependent luminescence lifetime (τ) and quantum yield (QY) of **1** in the solid-state

λ_{ex} / nm	325	340	355	370	385	400	415	430	432
τ_{616} / μs	478	478	480	477	489	483	445	428	413
QY / %	6.73	6.08	5.54	5.38	4.21	3.89	3.05	2.85	2.85

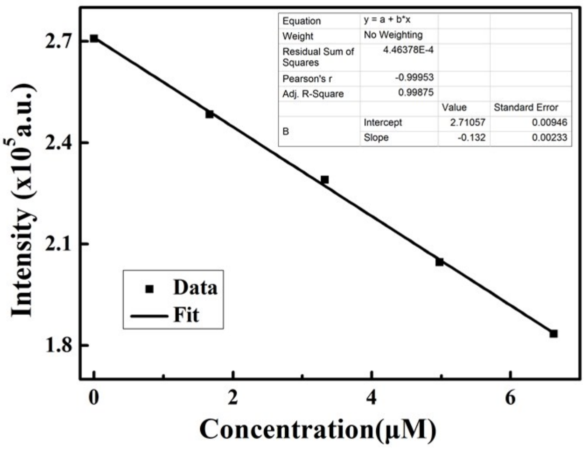

Fig. S6 Plot of luminescent intensity *versus* [bilirubin].

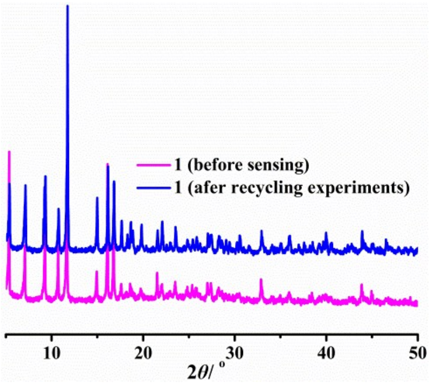

Fig. S7 PXRD patterns of **1** before and after five recycling experiments for bilirubin sensing.

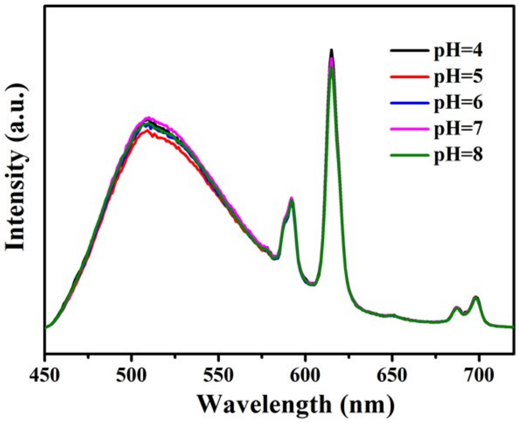

Fig. S8 Emission spectra of **1** dispersed in water with different pH values (λ_{ex} = 432 nm).

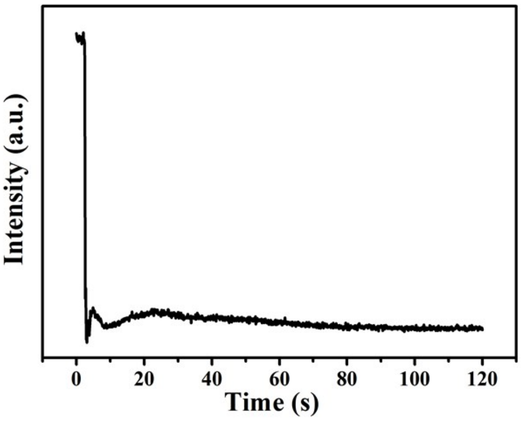

Fig. S9 Time-dependent fluorescence intensity of **1** at 616 nm upon introduction of bilirubin in an aqueous solution with pH = 7.4.

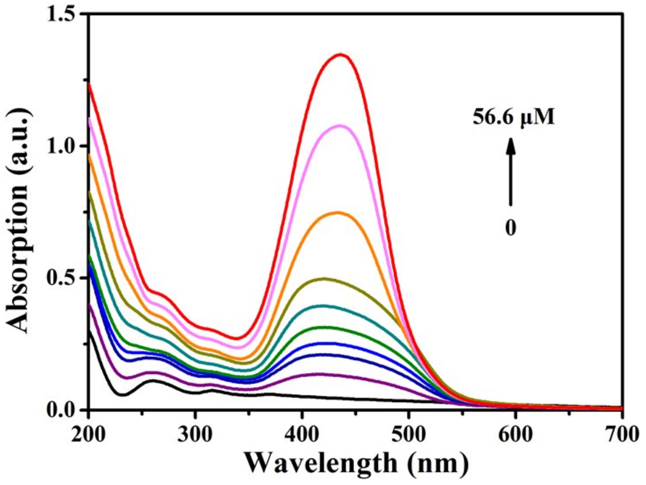

Fig. S10 UV-vis absorption spectra of **1** in the presence of different concentrations of bilirubin.

Table S3 Parameters used for the IFE correction of **1** in the presence of different concentrations of bilirubin

$C_{bilirubin}$ / μM	A_{ex}	A_{em}	I_{obsd}	I_{cor}	I_{cor}/I_{obsd}	E_{obsd} (%)	E_{cor} (%)
0	0.045	0.019	270870	290162	1.071	0	0
3.32	0.132	0.010	229035	267766	1.169	0.154	0.077
6.62	0.207	0.013	183430	233206	1.271	0.323	0.196
9.90	0.251	0.015	165645	221017	1.334	0.388	0.238
13.16	0.309	0.015	148530	210361	1.416	0.452	0.275
16.39	0.388	0.013	129360	197845	1.529	0.522	0.318
19.61	0.489	0.018	115330	195372	1.694	0.574	0.327
32.26	0.746	0.012	91680	193892	2.115	0.662	0.332
44.59	1.074	0.013	69800	190639	2.731	0.742	0.343
56.60	1.340	0.013	58230	190397	3.270	0.785	0.344

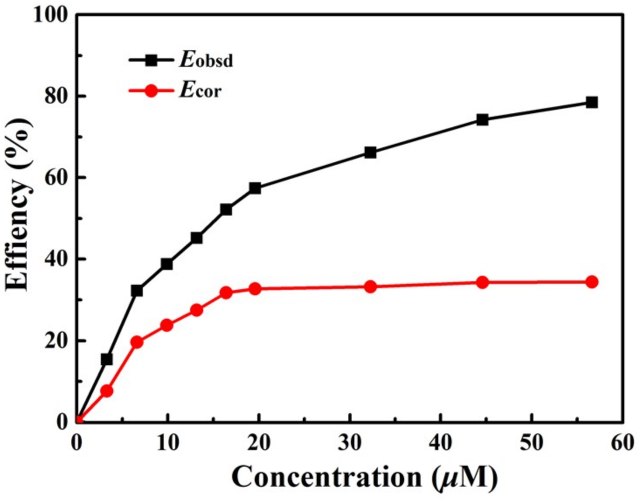

Fig. S11 Observed (E_{obsd}) and corrected (E_{cor}) fluorescence quenching efficiency of **1** after the addition of different concentrations of bilirubin.

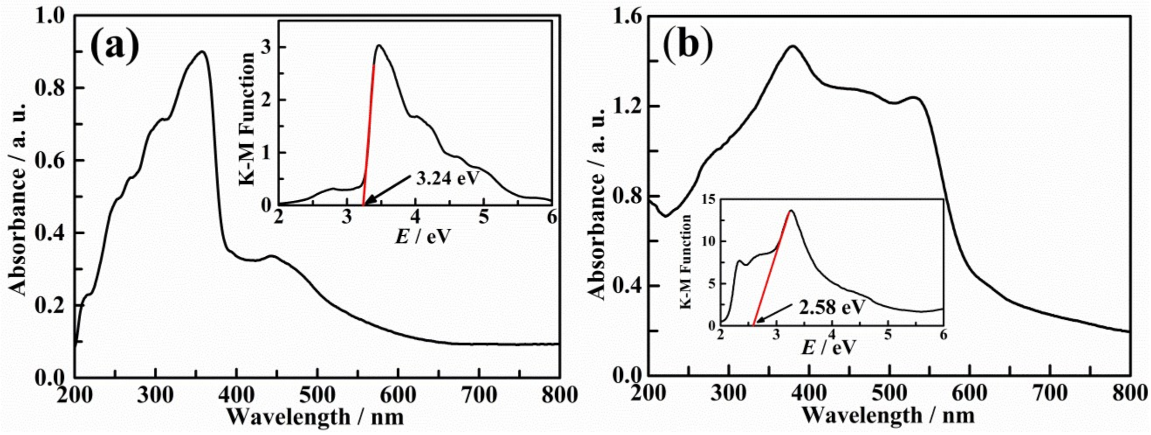

Fig. S12 UV-vis diffuse reflectance spectra of **1** (a) and bilirubin (b) (Inset: Plots of K-M function vs. energy for **1** and bilirubin).
